# Thermally Conductive AlN‐Network Shield for Separators to Achieve Dendrite‐Free Plating and Fast Li‐Ion Transport toward Durable and High‐Rate Lithium‐Metal Anodes

**DOI:** 10.1002/advs.202200411

**Published:** 2022-04-23

**Authors:** Yue Guo, Qiang Wu, Liwei Liu, Guochang Li, Lijun Yang, Xizhang Wang, Yanwen Ma, Zheng Hu

**Affiliations:** ^1^ Key Laboratory of Mesoscopic Chemistry of MOE and Jiangsu Provincial Laboratory for Nanotechnology School of Chemistry and Chemical Engineering Nanjing University Nanjing 210023 P. R. China; ^2^ Key Laboratory for Organic Electronics and Information Displays (KLOEID) & Institute of Advanced Materials (IAM) Jiangsu Key Laboratory for Biosensors Jiangsu National Synergetic Innovation Center for Advanced Materials (SICAM) Nanjing University of Posts and Telecommunications Nanjing 210023 P. R. China

**Keywords:** composite separators, dendrite‐free plating, high‐rate Li metal anodes, super electrolyte‐philic channels, thermally conductive AlN‐networks

## Abstract

Lithium‐metal anodes suffer from inadequate rate and cycling performances for practical application mainly due to the harmful dendrite growth, especially at high currents. Herein a facile construction of the porous and robust network with thermally conductive AlN nanowires onto the commercial polypropylene separator by convenient vacuum filtration is reported. The so‐constructed AlN‐network shield provides a uniform thermal distribution to realize homogeneous Li deposition, super electrolyte‐philic channels to enhance Li‐ion transport, and also a physical barrier to resist dendrite piercing as the last fence. Consequently, the symmetric Li|Li cell presents an ultralong lifetime over 8000 h (20 mA cm^−2^, 3 mAh cm^−2^) and over 1000 h even at an unprecedented high rate (80 mA cm^−2^, 80 mAh cm^−2^), which is far surpassing the corresponding performances reported to date. The corresponding Li|LiFePO_4_ cell delivers a high specific capacity of 84.3 mAh g^−1^ at 10 C. This study demonstrates an efficient approach with great application potential toward durable and high‐power Li–metal batteries and even beyond.

## Graphical abstract

A porous and robust network of thermally conductive aluminum nitride (AlN) nanowires is conveniently constructed onto the commercial polypropylene separator. Such an AlN‐network shield provides the uniform thermal distribution for homogeneous Li deposition and dendrite‐free plating thereof, super electrolyte‐philic channels for fast Li‐ion transport, and also a last physical barrier to resist dendrite piercing, leading to the unprecedented cycling performance at high rate.

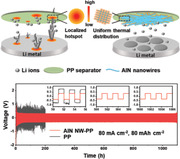

## 1 Introduction

The rapid development of portable electronics and electric vehicles demands power sources with higher energy densities.^[^ ^1^, ^2^ ^]^ Lithium‐ion batteries (LIBs), the predominant commercial power source, take the graphite anodes with a theoretical capacity of 372 mAh g^−1^, which usually deliver the energy density of <300 Wh kg^–1^.^[^ ^3^, ^4^ ^]^ To increase the energy density, one of the effective approaches is to replace the graphite anode with the advanced anodes with higher capacities.^[^ ^4^, ^5^ ^]^ After decades of searching, lithium metal returns to the researcher's horizon due to its ultrahigh theoretical capacity (3860 mAh g^−1^), the lowest electrochemical potential (‐3.04 V versus standard hydrogen electrode), as well as the potential use in next‐generation energy storage technologies such as Li–S and Li–air batteries.^[^ ^6^ ^]^ However, the long‐standing challenges such as the Li dendrite growth, large volume change, unstable solid–electrolyte interface, and low electrode kinetics have not been well addressed yet.^[^ ^7^, ^8^ ^]^ The Li dendrite growth arising from the inhomogeneous deposition/stripping of lithium during cycling is the most critical problem, which could puncture the separator to induce fatal safety hazards and is also associated with the other challenging issues aforementioned.^[^ ^9^, ^10^ ^]^ Especially at high currents, a large amount of Joule heat leads to severe nonuniform temperature distribution, which makes dendrite growth even serious at the local hotspots since the high temperature favors the fast Li deposition.^[^ ^11^, ^12^, ^13^ ^]^ Hence, the Li–metal anodes usually exhibit inferior rate and cycling capabilities.^[^ ^10^ ^]^ Suppressing the dendrite growth, especially at high currents, is imperative for developing advanced Li–metal anodes.

To date, the strategies to address the dendrite growth usually include: i) constructing the stable Li host with porous carbons,^[^ ^14^, ^15^, ^16^, ^17^, ^18^ ^]^ or metal frameworks;^[^ ^19^, ^20^, ^21^ ^]^ ii) optimizing the electrolyte by adding additives,^[^ ^22^, ^23^, ^24^ ^]^ using solid‐state electrolytes,^[^ ^25^, ^26^, ^27^ ^]^ etc.; iii) designing the composite separators via different modifications.^[^ ^28^, ^29^, ^30^, ^31^ ^]^ Among them, the separator modification has the advantages of easy operation, abundant material options, and multifunctional integration. In principle, an ideal composite separator should possess the merits of i) high thermal conductivity to achieve uniform temperature distribution and homogeneous Li deposition, ii) highly porous structure to enable fast Li‐ion transport, iii) high rigidity to resist dendrite piercing.

**Scheme 1:**
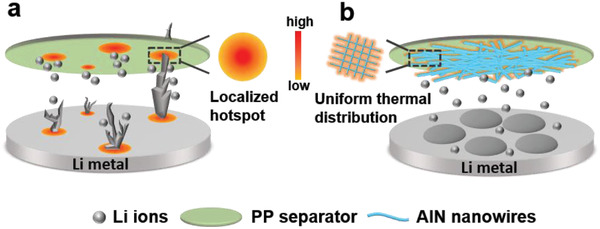
Schematic illustrations of Li deposition behavior. a) With blank PP separator, b) with AlN NW‐PP composite separator. With blank PP of poor thermal conductivity, the numerous localized hotspots are generated within batteries at high rates, leading to severe Li dendrite growth around these spots (a). With AlN NW‐PP of high thermal conductivity, a uniform thermal distribution is achieved, leading to homogeneous Li deposition and dendrite‐free plating thereof (b).

Aluminum nitride (AlN) has excellent thermal conductivity (319 W m^−1^ K^−1^),^[^ ^32^ ^]^ high rigidity (23.7 GPa),^[^ ^33^ ^]^ and electrochemical stability to Li metal.^[^ ^34^, ^35^ ^]^ Its porous network could be an ideal shield for separators to stabilize Li anodes. Herein, we report a facile construction of the porous and robust network with thermally conductive AlN nanowires onto the commercial polypropylene (PP) separator by convenient vacuum filtration. The so‐constructed composite separator of AlN‐network and PP, denoted as AlN NW‐PP, can not only achieve a uniform thermal distribution for homogeneous Li deposition and dendrite‐free plating thereof but also provide super electrolyte‐philic channels for fast Li‐ion transport, much superior to the counterpart of blank PP separator, as illustrated in **Scheme** **1**. As a result, the symmetric Li|Li cell with the AlN NW‐PP separator demonstrates an unprecedented performance, with an ultralong reversible Li plating/stripping over 8000 h under a high current density of 20 mA cm^−2^ with a capacity of 3 mAh cm^−2^, and over 1000 h at an ultrahigh current density of 80 mA cm^−2^ with a high capacity of 80 mAh cm^−2^. The Li|LiFePO_4_ cell with the AlN NW‐PP presents a high specific capacity of 84.3 mAh g^−1^ at the high rate of 10 C and superior cycling stability, demonstrating great potential applications.

## 2 Results and Discussion

**Figure 1:**
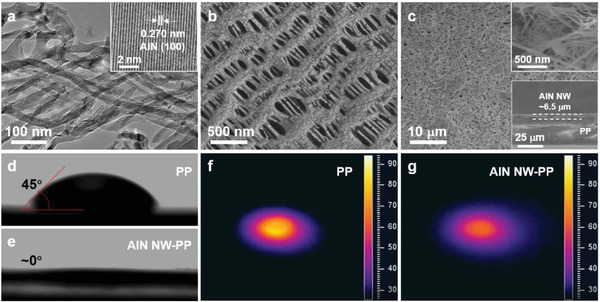
Characterizations of AlN nanowires, blank PP separator, and AlN NW‐PP separator. a) Transmission electron microscopy (TEM) image of AlN nanowires. Inset is the corresponding high‐resolution TEM image. Top‐view scanning electron microscopy (SEM) images of b) the blank PP and c) AlN NW‐PP, respectively. In (c), the upper inset is a local enlargement, and the bottom inset is the cross‐section SEM image. The contact angle of the DOL/DME (1,3‐dioxolane and 1,2‐dimethoxyethane) electrolyte on d) the blank PP and e) the AlN NW‐PP. The temperature distributions for f) the blank PP and g) AlN NW‐PP.

The AlN nanowires were prepared by thermal nitridation of aluminum powder at 1200 °C,^[^ ^36^ ^]^ and then the AlN nanowires slurry was coated on the PP separator by convenient vacuum filtration to form the AlN NW‐PP separator (see Experimental Section in Supporting Information). The characterizations on the AlN nanowires and AlN NW‐PP are shown in **Figure** **1**. The AlN nanowires have a diameter of ≈20 nm and a length of a few tens of micrometers with high crystallinity (Figure 1a; Figure S1, Supporting Information). For the AlN NW‐PP, the big holes in the blank PP were covered by the interlaced AlN nanowires, which formed the porous network on the PP surface with a thickness of ≈6.5 µm (Figure 1b,c). The AlN NW‐PP composite separator demonstrates a super electrolyte‐philic character with a contact angle approaching 0°, i.e., with much better affinity to the electrolyte than the PP separator with a contact angle of ca. 45°, which should be associated with the unique micro/nanostructures and surface chemical polarity of AlN (Figure 1d,e).^[^ ^37^ ^]^ Such a super electrolyte‐philic network much promotes the diffusion/transport of ions on the interfaces.^[^ ^38^, ^39^ ^]^ Besides, the interaction between the Lewis acid sites on AlN and the anions of electrolyte could release more “free” Li‐ions, which also favors the increase of the Li^+^ transference number (*t*_Li_^+^).^[^ ^39^, ^40^ ^]^ Consequently, the *t*_Li_^+^ for the AlN NW‐PP separator reaches 0.51, much larger than 0.37 for the PP separator (Figure S2, Supporting Information). The high rigidity of the AlN NW can prevent the PP separator of low mechanical strength from being punctured by Li dendrites, just in case. The porous network could also alleviate the strain arising from the volume change of the Li anode during cycling.^[^ ^9^ ^]^

More importantly, the AlN NW‐PP is beneficial to the heat dissipation and uniform temperature distribution owing to the high thermal conductivity of AlN. By heating the AlN NW‐PP or PP separator with an infrared laser and recording the temperature distribution with an infrared camera, the temperature distribution was obtained. The PP separator had a bright central hotspot (≈90 °C) with a large temperature gradient, which will exacerbate the Li dendrite growth at the hotspots (Figure 1f). In contrast, the central temperature of the AlN NW‐PP was much reduced to 60 °C with a rather uniform temperature distribution, which favors the homogeneous Li deposition and effectively suppresses the formation of Li dendrites thereof (Figure 1g).^[^ ^11^ ^]^ Worthy of mentioning is that the resulting AlN NW‐PP separator can be twisted or rolled without cracking and shedding, exhibiting excellent flexibility (Figure S3, Supporting Information). According to the preceding characterization results, the AlN NW‐PP separator should be superior to the blank PP in suppressing Li dendrite growth and piercing and facilitating the Li‐ion transport, which could much boost the electrochemical performance of the Li anodes.

**Figure 2:**
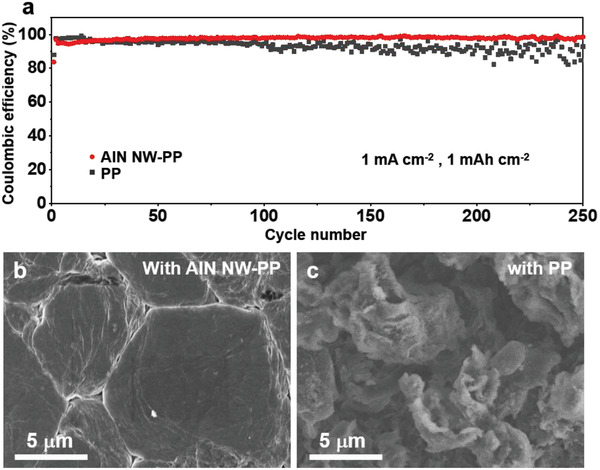
Characterizations on Li|Cu cells with AlN NW‐PP or PP separator. a) Coulombic efficiencies at 1 mA cm^–2^ with a capacity of 1 mAh cm^–2^. b,c) SEM images of the Li deposition on Cu electrodes after 250 cycles plus one additional plating.

The Li|Cu cells with the AlN NW‐PP or PP separator, denoted as Li|AlN NW‐PP|Cu or Li|PP|Cu, respectively, were charged/discharged at 1 mA cm^–2^ with an areal capacity of 1 mAh cm^–2^ to examine the effect of separator on the cycling stability of the Li metal. **Figure** **2** displays the evolution of Coulombic efficiency (CE) during cycling and the morphology of Li deposition on the Cu electrode after cycling. As expected, the Li|AlN NW‐PP|Cu cell presents a steady CE with a high value of 98.5% after 250 cycles. In contrast, the CE of the Li|PP|Cu cell drops to 87.3% after 130 cycles, followed by a large fluctuation. The low CE reflects the irreversible Li plating/stripping, and the large fluctuation can be attributed to the fracture of Li dendrites and the “repair” of the fragments produced in previous cycles (Figure 2a).^[^ ^41^ ^]^ The overpotentials of the Li|AlN NW‐PP|Cu are smaller and more stable than those of the Li|PP|Cu during Li plating and stripping, again showing the superiority of the AlN NW‐PP separator (Figure S4, Supporting Information). After 250 cycles plus one additional plating, the flat and compact Li film absent from the dendrites formed on the Cu electrode in the Li|AlN NW‐PP|Cu, much different from the loose and dendritic Li deposition in the Li|PP|Cu (Figure 2b,c; Figure S5, Supporting Information). The dense Li film is beneficial to reduce the side reactions and the “dead” lithium, which is responsible for the high CE of the Li|AlN NW‐PP|Cu.^[^ ^9^ ^]^ When the current density increased to 2.0 and 5.0 mA cm^−2^, the Li|AlN NW‐PP|Cu still remained high CE of 98.4% and 95.7% after 150 cycles with little fluctuation, much better than the inferior CE with big fluctuation for the Li|PP|Cu (Figure S6, Supporting Information). We further tested the CE of Li|Cu cells with an alternative, which demonstrated a higher average CE and smaller overpotential for the Li|AlN NW‐PP|Cu than the Li|PP|Cu (Figure S7, Supporting Information).^[^ ^42^ ^]^

**Figure 3:**
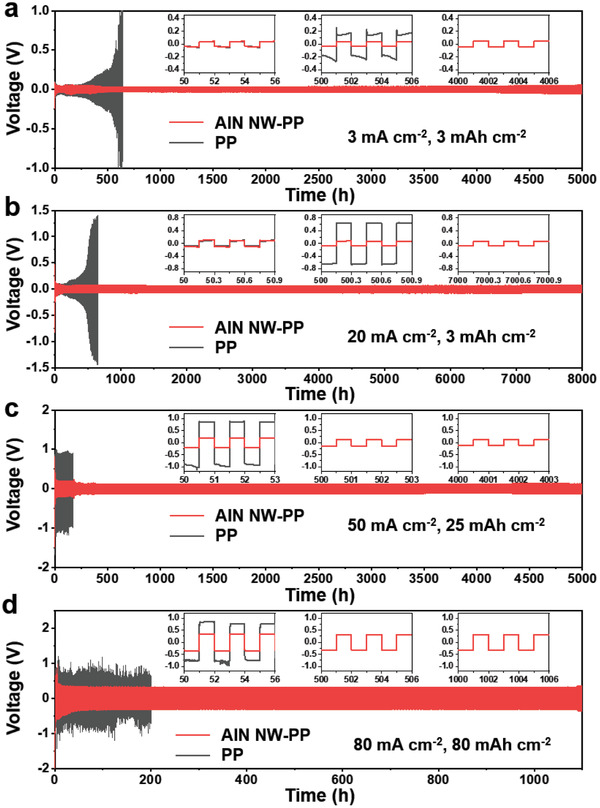
Electrochemical performances of symmetric Li|Li cells with AlN NW‐PP or PP separator at different conditions. a) 3 mA cm^−2^, 3 mAh cm^−2^. b) 20 mA cm^−2^, 3 mAh cm^−2^. c) 50 mA cm^−2^, 25 mAh cm^−2^. d) 80 mA cm^−2^, 80 mAh cm^−2^. Insets in (a–d) are the local magnifications.

The symmetric Li|Li cells with the AlN NW‐PP or PP separator denoted as Li|AlN NW‐PP|Li or Li|PP|Li, respectively, were assembled to further reveal the effect of the AlN NW on the cycling performance of the Li–metal electrode, as shown in **Figure** **3**. Clearly, the Li|AlN NW‐PP|Li exhibited much lower overpotentials and much longer cycling life than the Li|PP|Li in all the test conditions. Specifically, the overpotential of the Li|PP|Li cell exponentially increases to over 500 mV for no more than 600 h when cycled at 3 mA cm^–2^ with the capacity of 3 mAh cm^–2^ (Figure 3a), which may result from the near depletion of electrolyte due to the gradually accumulated dendrites and “dead” Li.^[^ ^28^ ^]^ In contrast, the Li|AlN NW‐PP|Li cell exhibits small overpotentials (35 mV@50 h, 38 mV@500 h, and 42 mV@4000 h) and relatively steady voltage plateaus with long‐term cycling stability over 5000 h (Figure 3a). The corresponding electrochemical impedance spectroscopies (EIS) show the close resistances at the initial stage for the two cells. Once cycled, the resistance of the Li|AlN NW‐PP|Li is lower than that of the Li|PP|Li with the same cycles, and the former increases much more slowly than the latter, indicating the inhibited Li dendrites and less electrolyte depletion for the former (Figure S8, Supporting Information). As the current density increases to 20 mA cm^−2^, the overpotential of the Li|PP|Li goes beyond 500 mV within 500 h, while the Li|AlN NW‐PP|Li is charged/discharged for over 8000 h with a stable voltage hysteresis (97 mV@50 h, 74 mV@500 h, and 78 mV at 7000 h) (Figure 3b). Even at an ultrahigh current density of 50 mA cm^−2^ with a high capacity of 25 mAh cm^−2^, the Li|AlN NW‐PP|Li can still maintain the relatively low overpotential over 5000 h (203 mV at 50 h, 130 mV at 500 h, and 127 mV at 4000 h), indicating its stable Li plating/stripping, much better than the Li|PP|Li with a very large overpotential (≈1 V at 50 h) (Figure 3c). With the increase of the current density, the overpotentials of the Li|PP|Li cells presented an obvious increase due to the limited Li^+^ transport and increased side effects. In contrast, the overpotentials of the Li|AlN NW‐PP|Li cells increased more slowly, which can be attributed to the significantly improved Li^+^ transport and stabilized solid electrolyte interface (SEI) due to the high Li^+^ transference number and uniform Li deposition. To evaluate the ultimate potential of the AlN‐network shield for separators, the Li|AlN NW‐PP|Li under a harsh condition of 80 mA cm^−2^/80 mAh cm^−2^ were tested. The Li|AlN NW‐PP|Li manifests an unprecedented cycling performance over 1000 h with a stable voltage hysteresis (332 mV at 50 h, 325 mV at 500 h, and 325 mV at 1000 h) (Figure 3d). Such a long lifetime at such a large current density and capacity sets a new record, far surpassing the best performance of Li–metal batteries to date (Table S1, Supporting Information). The overpotentials of the Li|AlN NW‐PP|Li cells gradually reduce in the initial cycles and then stabilize, which could be attributed to the SEI formation on the AlN NW attaching the Li foil.^[^ ^29^, ^43^ ^]^ With the commercial carbonate‐based electrolytes (i.e., 1 m LiPF_6_ in EC/DMC), the Li|AlN NW‐PP|Li cell also demonstrates much superior performance to the Li|PP|Li cell (Figure S9, Supporting Information).

**Figure 4:**
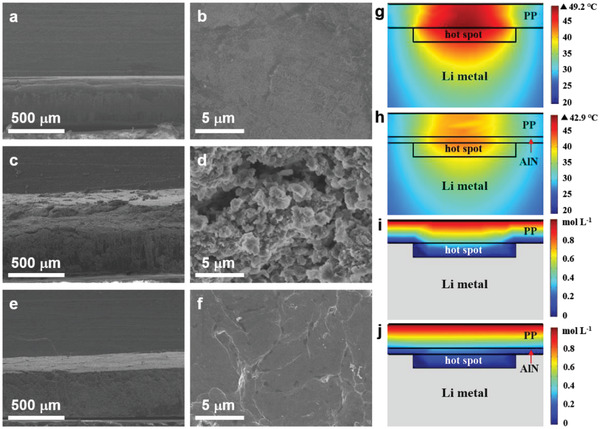
SEM images of Li–metal electrodes and finite element simulations. a,b) The pristine Li metal electrode. The Li metal electrodes of c,d) the Li|PP|Li and e,f) the Li|AlN NW‐PP|Li after 600 h cycling under 20 mA cm^−2^. Note: a,c,e) are the side‐view and b,d,f) are the top‐view images. g–j) Finite element simulations of g,h) the temperature distribution and i,j) the Li^+^ concentration distribution.

After 600 h cycling test at 20 mA cm^−2^, the Li–metal electrodes of the Li|Li cells were examined to clarify the influence of separators, as shown in **Figure** **4**. The pristine Li metal had a smooth surface and a compact structure (Figure 4a,b). After cycling in the Li|PP|Li cell, the volume of Li anode was much expanded, and numerous Li dendrites were formed on the Li–metal electrode, showing a rough and loose structure (Figure 4c,d). In contrast, the Li–metal electrode of the Li|AlN NW‐PP|Li cell was flat and dense, demonstrating a dendrite‐free plating on the surface as expected (Figure 4e,f). The obvious morphologic difference indicates that the AlN NW‐PP separator effectively promotes the homogeneous Li deposition and inhibits the formation of Li dendrites (Figure S10, Supporting Information). Finite element analysis indicates that the cell with the AlN NW‐PP has a lower peak temperature and a smaller temperature gradient around the hotspot than that with the PP, which is due to the high thermal conductivity of AlN (Figure 4g,h; Figure S11, Supporting Information). The corresponding Li^+^ concentration distribution presents the smaller horizontal concentration gradient on the surface of Li anode for the former (Figure 4i,j), which effectively promotes the homogeneous Li deposition, consistent with the different morphologies of Li metal after plating (Figure 4c–f).

The preceding results demonstrate that the Li|AlN NW‐PP|Li cells exhibit the unprecedented long stripping/plating lifetime under ultrahigh current densities, much better than the case of the Li|PP|Li cells. The remarkable performance can be ascribed to the thermally conductive and robust AlN NW shield on PP, which can suppress the formation of hotspots arising from the Joule heating and thus restrain the inhomogeneous Li dendrites growth. Together with the facilitated Li‐ion transport due to the super electrolyte‐philic network and the resistance to the piercing of dendrites due to the toughness of AlN, the superior electrochemical performance of Li–metal anodes is achieved.

**Figure 5:**
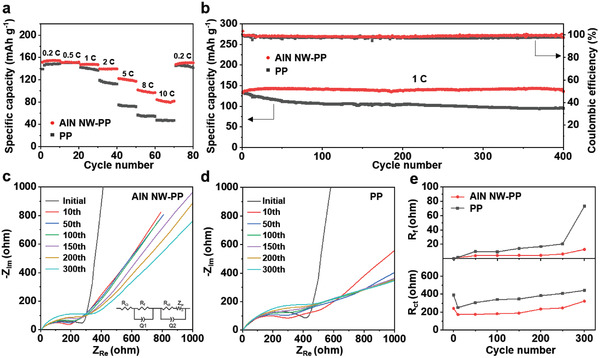
Electrochemical performances of Li|LiFePO_4_ cells with AlN NW‐PP or PP separator. a) Rate performances. b) Long‐term cycling stabilities at 1 C (1 C = 170 mA g^−1^). c,d) EIS at initial and after different cycles with c) AlN NW‐PP or d) PP separator. Inset in (c) is the equivalent circuit. e) Variations of *R*_f_ and *R*_ct_ with cycle number. Note: The sharp decrease of *R*_ct_ after the first cycle corresponds to the activation of cells.^[^ ^44^ ^]^

To evaluate the application potential, the Li|LiFePO_4_ cells with the AlN NW‐PP or PP separator were tested (**Figure** **5**; Figure S12, Supporting Information). The Li|AlN NW‐PP|LiFePO_4_ cell displays the high specific capacities of 154.7, 151.5, 147.6, 139.4, 122.2, 101.8 mAh g^–1^ at 0.2, 0.5, 1, 2, 5, 8 C, and still achieves 84.3 mAh g^–1^ even at a high rate of 10 C. In contrast, Li|PP|LiFePO_4_ cell delivers much lower capacities especially at high rates (Figure 5a). Specifically, at the small current densities, both the AlN NW‐PP and PP separators can meet the requirement for the Li‐ion transport, leading to the close specific capacities, i.e., only a slight improvement for the former. With increasing the current density, the AlN NW‐PP separator can basically keep up with the demand for fast Li‐ion transport kinetics, while the PP separator cannot, due to the larger Li^+^ transference number (*t*_Li_^+^) for the former (0.51) than the latter (0.37). As a result, the capacity improvement becomes more and more evident at high current densities. In addition, at small current densities, the inhomogeneity of temperature distribution is not so severe for both the AlN NW‐PP and the PP due to the limited Joule heating, leading to the relatively homogenous Li‐ion transport with close specific capacities. At high current densities, the large amounts of Joule heating cause severe nonuniform temperature distribution on the PP, leading to inhomogeneous Li‐ion transport. In contrast, the situation is much better on the AlN NW‐PP thanks to its high thermal conductivity, leading to rather homogeneous Li‐ion transport and increased specific capacities.

The long‐term cycling tests indicate that the Li|AlN NW‐PP|LiFePO_4_ cell delivers a specific capacity of 136 mAh g^−1^ at 1 C over 400 cycles with a high capacity retention of 94.8% and a steady CE of nearly 100%. For the Li|PP|LiFePO_4_ cell, the specific capacity fades to 95.2 mAh g^−1^ over 400 cycles with the slightly lower CE (Figure 5b). At a higher current density of 3 C, the Li|AlN NW‐PP|LiFePO_4_ cell retains 104.4 mAh g^−1^ over 300 cycles, whereas only 76.4 mAh g^−1^ for the Li|PP|LiFePO_4_ cell, with the lower potential gaps between the discharge and charge plateaus for the former (Figure S13, Supporting Information). When the areal loading of LiFePO_4_ was increased to 6 mg cm^−2^, the Li|AlN NW‐PP|LiFePO_4_ cell still exhibited more stable long‐term cycling and higher rate performance than the Li|PP|LiFePO_4_ cell (Figure S14, Supporting Information). To get a deep insight into the cycling stability, the evolution of charge transfer kinetics for the two Li|LiFePO_4_ cells upon cycling was examined by EIS. By simulating with the equivalent circuit, the contact resistance (*R*_Ω_), interfacial resistance of SEI film (*R*_f_), and charge transfer resistance (*R*_ct_) were obtained. The *R*_f_ values of the Li|AlN NW‐PP|LiFePO_4_ cell are lower and increase more slowly than the corresponding ones of the Li|PP|LiFePO_4_ cell, indicating the better stability of the SEI for the former during cycling. The *R*_ct_ values of the former are also lower than that of the latter, showing the better charge transfer kinetics of the former (Figure 5c–e; Figure S15, Supporting Information). We also performed the X‐ray photoelectron spectroscopy (XPS) for the cycled Li anodes of the two cells to understand the influence of SEI composition on the battery performance, and quite similar XPS spectra were obtained except for the F1s. More LiF and Li_x_PF_y_ species were detected on the cycled Li anode of the Li|AlN NW‐PP|LiFePO_4_ cell (Figure S16, Supporting Information). The F‐containing species in the SEI film originated from the decomposition of LiPF_6_ with the promotion of Lewis acid sites on AlN nanowires.^[^ ^45^ ^]^ Such a SEI film could promote the transport and homogeneous deposition of Li^+^ on the Li anode,^[^ ^46^ ^]^ thus beneficial to the improvement of the rate and cycling performances. The promotion of AlN NW‐PP to the cycling performance of LiFePO_4_|Li‐plated Cu cells was also evaluated. The cell with PP separator exhibited a discharge capacity of 147.5 mAh g^‐1^ in the first cycle and a very low capacity of 7.4 mAh g^‐1^ after 80 cycles, accompanied by the fast decay of CE. In contrast, the cell with AlN NW‐PP separator delivered an initial capacity of 151.2 mAh g^‐1^ and a high retained capacity of 146.9 mAh g^‐1^ after 80 cycles, with a stable CE of ca. 100% (Figure S17, Supporting Information). In total, the separator modification with thermally conductive and porous AlN‐network can promote homogeneous Li deposition and Li‐ion transport, which guarantees the stable SEI and improves charge transfer kinetics, therefore leads to the better cycling performance of Li–metal batteries.

## 3 Conclusion

In summary, taking advantage of the high thermal conductivity and rigidity of AlN nanowires, we have successfully constructed a porous robust network shield for the commercial PP separator by a convenient vacuum filtering. For the so‐constructed composite separator of AlN NW‐PP, the high thermal conductivity leads to the uniform thermal distribution. The unique micro/nanostructures of AlN‐network and surface chemical polarity of AlN lead to the superior electrolyte affinity. Consequently, the AlN NW‐PP can not only boost a homogeneous Li deposition to approach a dendrite‐free anode, as supported by the finite element simulation results, but also facilitate the Li‐ion transport, as reflected by the much larger Li^+^ transference number of 0.51 than 0.37 for the PP separator. The robust network could resist the piercing of dendrites as the last fence and meanwhile alleviate the strain of volume change during cycling. As a result, with the AlN NW‐PP, the Li|Li cells demonstrate an ultralong lifetime over 8000 h (20 mA cm^−2^, 3 mAh cm^−2^) and over 1000 h even at the unprecedented high rate (80 mA cm^−2^, 80 mAh cm^−2^). The corresponding Li|LiFePO_4_ cell delivers a high specific capacity of 84.3 mAh g^−1^ at 10 C. When employed in Na–metal anode, the AlN NW‐PP also shows advantages in Na plating/stripping with a stable cycling performance over 600 h at 5 mA cm^−2^, totally different from the quick failure for the case with the PP (Figure S18, Supporting Information). In principle, the AlN NW‐PP could also be effective for the other metal anodes beset by dendrite growth such as K or Zn anodes, and the AlN network could also be substituted by some other rigid thermally conductive networks such as diamond‐like carbon, BN, and Si_3_N_4_, which suggests a general strategy toward durable and high‐rate metal anodes.

## Conflict of Interest

The authors declare no conflict of interest.

## Supporting information

Supporting Information

## Data Availability

The data that support the findings of this study are available from the corresponding author upon reasonable request.
