# Supplementary material for: Thermally Conductive AlN‐Network Shield for Separators to Achieve Dendrite‐Free Plating and Fast Li‐Ion Transport toward Durable and High‐Rate Lithium‐Metal Anodes
Source: Adv Sci (Weinh). 2022 Apr 23;9(18):2200411. doi: 10.1002/advs.202200411 (PMC9218647; doi:10.1002/advs.202200411)
Supplement: Supplementary file 1 — Supporting Information [file ADVS-9-2200411-s001.pdf]

ADVANCED SCIENCE
Open Access

Supporting Information

for *Adv. Sci.*, DOI 10.1002/advs.202200411

Thermally Conductive AlN-Network Shield for Separators to Achieve Dendrite-Free Plating and Fast Li-Ion Transport toward Durable and High-Rate Lithium-Metal Anodes

Yue Guo, Qiang Wu, Liwei Liu, Guochang Li, Lijun Yang, Xizhang Wang, Yanwen Ma and Zheng Hu**

Supporting Information

Thermally-Conductive AlN-Network Shield for Separators to Achieve Dendrite-Free Plating and Fast Li-Ion Transport toward Durable and High-Rate Lithium-Metal Anodes

Yue Guo, Qiang Wu, Liwei Liu, Guochang Li, Lijun Yang, Xizhang Wang, Yanwen Ma, and Zheng Hu**

Experimental Section

Preparation of AlN nanowires and composite separators: AlN nanowires were prepared on a large scale by nitriding Al powder at 1200 °C in the N_2/NH_3 (NH_3, 4%) flow, similar to our previous study.[1] The AlN nanowires and polyvinylidene difluoride (PVDF) binder were mixed in N-methyl-2-pyrrolidinone (NMP) with a mass ratio of 9:1 to form a homogeneous slurry. Then, the slurry was coated on a PP separator (Celgard 2400) by convenient vacuum filtration, followed by vacuum drying at 50 °C for 10 h. The loading of AlN nanowires was ca. 0.5 mg cm^{-2}.

Sample Characterizations: SEM (Hitachi S4800), high-resolution TEM (JEM-2100), and X-ray diffraction (Philips X'pert Pro X-ray diffractometer) were used to characterize the morphology and structure of samples. The contact angle measurements were carried out on a contact angle analyzer (OCA30, Data Physics Instruments GmbH). The temperature distribution was measured using a Fotric 225 infrared (IR) thermal camera (Fotric, Shanghai, China). X-ray photoelectron spectroscopy (XPS) was measured on Thermo ESCALAB 250Xi.

Fabrication of Li|Cu cells, symmetric Li|Li cells, and symmetric Na|Na cells: The CR2032-type cells were assembled in an Ar-filled glove box. For the Li|Cu cell, the AlN-coated side of separator faces the Cu foil. For the symmetric Li|Li cell and Na|Na cell, the separator was coated with the AlN-network on both sides. Two types of electrolytes were employed for Li deposition, i.e., 1.0 M lithium bis (trifluoromethane sulfonyl) imide (LiTFSI) in a mixture of 1,3-dioxolane (DOL) and 1,2-dimethoxyethane (DME) (1:1 by volume) with 2 wt% $LiNO_3$ additive, and 1.0 mol L^{-1} lithium hexafluorophosphate ($LiPF_6$) in a mixture of ethylene carbonate (EC) and dimethyl carbonate (DMC) (1:1 by volume), respectively. The electrolyte for Na deposition was 1.0 M $NaClO_4$ in tetraglyme.

Fabrication of Li|LiFePO$_4$ cells and *LiFePO$_4$|Cu cells:* To prepare the $LiFePO_4$ electrode, $LiFePO_4$ (Shanghai Macklin Biochemical Co., Ltd.), acetylene black, and PVDF with a mass ratio of 8:1:1 were mixed in NMP to form a homogeneous slurry and then spread on an Al foil, followed by vacuum drying at 80 °C for 12 h. The areal loading of $LiFePO_4$ was ~2.0 mg cm^{-2} and 6.0 mg cm^{-2}, respectively. The Li|$LiFePO_4$ cell was assembled by attaching the AlN-coated side of separator on the Li foil. For the $LiFePO_4$|Cu cell, the AlN-coated side of separator faces the Cu foil and 3 mAh cm^{-2} of Li was plated on Cu foil in advance. 50 µL electrolyte (1.0 mol L^{-1} $LiPF_6$ in an EC/DMC mixture) was used in all cells.

Electrochemical measurements: The electrochemical cycling tests were performed on a Land CT2001 battery tester. Electrochemical impedance spectroscopy (EIS) was recorded on VMP3 electrochemical workstation (Bio-logic) by applying an AC amplitude of 5.0 mV over a frequency ranging from 0.01 Hz to 1 MHz. Cyclic voltammetry (CV) was performed on VMP3 electrochemical workstation (Bio-logic) in a voltage range of 2.5~4.2 V at a scan rate of 0.1 mV s^{-1}. The Li^+ transference number (t_{Li^+}) was measured in the symmetric Li|Li cells. A chronoamperometry test with the ΔV of 10 mV was applied until a steady current (I_s) was obtained. The initial (I_0) and steady (I_s) currents that flow through the cell were measured. Meanwhile, the EIS was recorded from 0.01 Hz to 1 MHz with an oscillating potential of 10 mV before and after the chronoamperometry test. The initial (R_0) and final (R_s) interfacial resistances were obtained, respectively. t_{Li^+} was calculated by the following equation:[2]

$$t_{Li^+} = [I_s \times (\Delta V - I_0 \times R_0)] \,/\, [I_0 \times (\Delta V - I_s \times R_s)] \quad (1)$$

The Coulombic efficiency (CE) test was conducted at 1 mA cm^{-2}, 2 mA cm^{-2}, and 5 mA cm^{-2}, respectively, under a capacity of 1 mAh cm^{-2} with a stripping cut-off voltage of 1 V. We further tested the average CE of Li|Cu cells with an alternative reported by Zhang et al.[3] Specifically, 4 mAh cm^{-2} of Li was plated on the Cu substrate and then fully stripped to 1 V prior to depositing the Li reservoir (Q_T = 4 mAh cm^{-2}) at 0.4 mA cm^{-2}. The cell was plated and stripped with a capacity of 0.5 mAh cm^{-2} (Q_C) for n cycles at 0.4 mA cm^{-2}, followed by the final stripping (Q_S) performed at 0.4 mA cm^{-2} to 1 V.

Finite element simulation: COMSOL Multiphysics software (finite-element method) is employed to simulate the temperature distribution and Li^+ concentration distribution with and without the AlN layer by combining the "Heat Transfer in Solid" module, "Electric Currents" module, and "Transport of Diluted Species Physics Interfaces" module.[4]

Model description: A three-dimensional (3D) spatial model with Li metal anode (500 μm thick, 7 mm in radius), pristine PP separator (25 μm thick, 7 mm in radius), and AlN layer (6.5

μm thick, 7 mm in radius) was constructed. Li-ions are concentrated at the top of the separator and transferred to the surface of the Li-metal anode by the packed electrolytes. Six heat sources (15 μm in thickness, 100 μm in radius) are created on the Li foil to represent the hotspots generated during cycling (Figure S9).

The heat conduction is governed by the Fourier's Law and stationary conservation of energy.

$$q = -k\nabla T \tag{2}$$

$$\nabla \cdot q = Q \tag{3}$$

Where T is the temperature, q is the heat flux, k is the thermal conductivity, and Q is volumetric heat generation.

The flux of Li^+ is governed by the Nernst-Planck equation.

$$N_i = -D_i \nabla c_i - z_i \mu_i F c_i \nabla U \tag{4}$$

Where N_i denotes the transport vector (mol m^{-2} s^{-1}), c_i the concentration in the electrolyte (mol m^{-3}), z_i the charge for the ionic species, μ_i the mobility of the charged species (m^2 s^{-1} J^{-1} mol^{-1}), F Faraday's constant (A s mol^{-1}), and U the potential (V) in the electrolyte.

Boundary conditions: The concentration of the top and bottom boundary is set to 1 mol L^{-1} and 0 mol L^{-1}. The current density is set to 20 mA cm^{-2}, and the bottom Li electrode is grounded, i.e., U = 0 V. The diffusion coefficient of Li-ions in the electrolyte is set to 3.2×10^{-6} cm^2 s^{-1}.

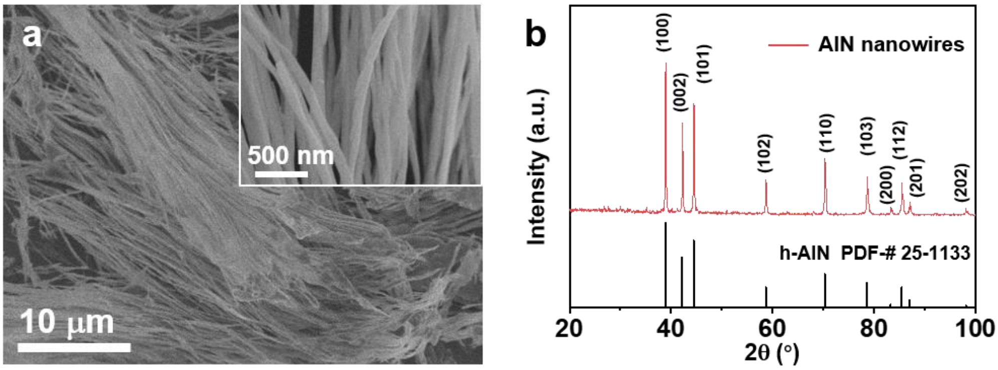

Figure S1. Characterizations of AlN nanowires. (a) SEM images. Inset is the local enlargement. (b) XRD pattern.

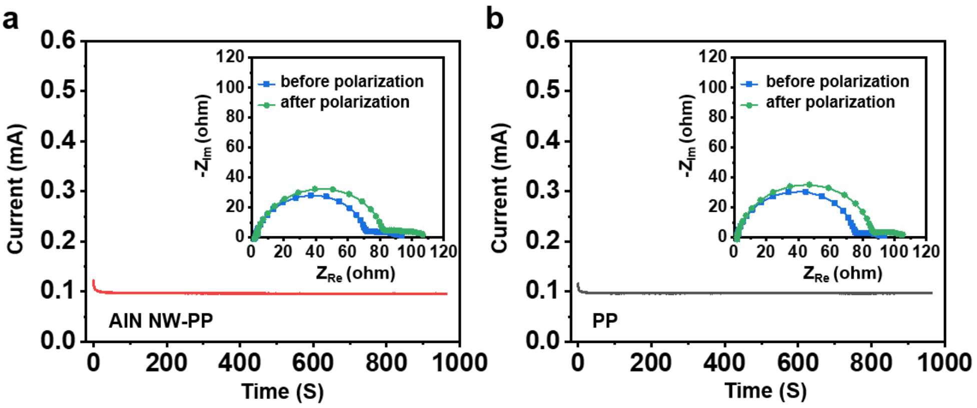

c

Separators	$I_{0(t=0)}$ (mA)	$I_{s(t=\infty)}$ (mA)	$R_{0(t=0)}$ (Ω)	$R_{s(t=\infty)}$ (Ω)	t_{Li^+}
AlN NW-PP	0.122	0.095	68.51	79.14	0.51
PP	0.116	0.096	78.45	83.37	0.37

Figure S2. Measurements of the Li^+ transference number (t_{Li^+}). (a,b) Chronoamperometries of the symmetric Li|Li cells with AlN NW-PP (a) or PP (b). Insets are EIS before and after polarization. (c) The corresponding parameters obtained from Figure S2a,b.

The Li^+ transference number (t_{Li^+}) was measured in the symmetric Li|Li cells. A chronoamperometry test with the ΔV of 10 mV was applied until a steady current (I_s) was obtained. The initial (I_0) and steady (I_s) currents that flow through the cell were measured. Meanwhile, the EIS spectra were recorded from 0.01 Hz to 1 MHz with an oscillating potential of 10 mV before and after the chronoamperometry test. The initial (R_0) and final (R_s) interfacial resistances were obtained, respectively. The t_{Li^+} values (Figure S2c) were calculated by the equation: $t_{Li^+} = [I_s\times(\Delta V - I_0\times R_0)]/[I_0\times(\Delta V - I_s\times R_s)]$.[2]

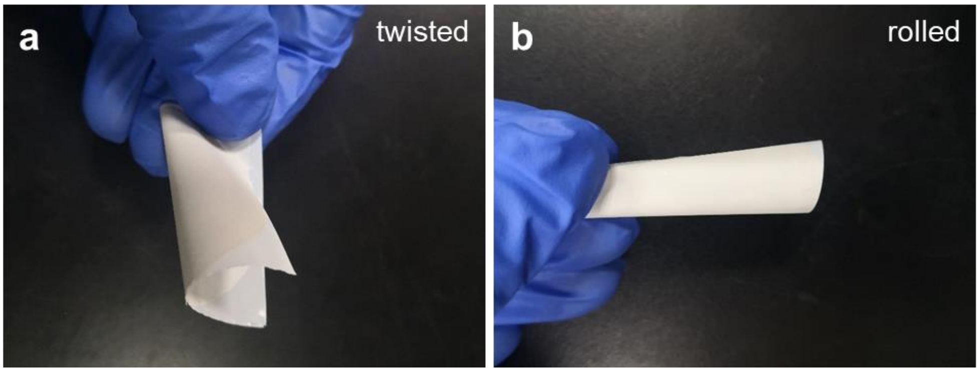

Figure S3. Photographs of the AlN NW-PP separator. (a) twisted, (b) rolled.

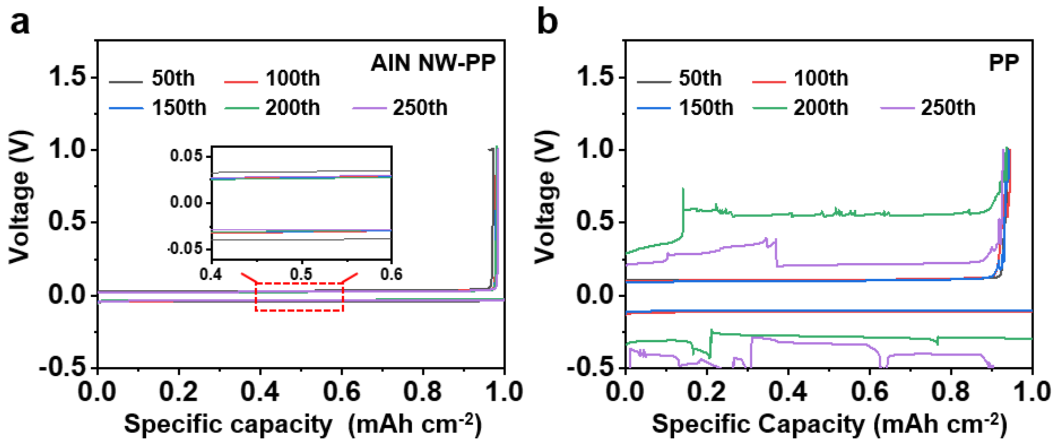

Figure S4. Charge and discharge curves of Li plating/stripping processes in Li|Cu cells with different separators. (a) AlN NW-PP, (b) PP. Inset in (a) is the local enlargement.

The Li|AlN NW-PP|Cu exhibits small and steady polarization voltage during the whole test. In contrast, the polarization voltage of the Li|PP|Cu is much larger, and the voltage fluctuates violently at 200th and 250th cycle, which can be ascribed to the much increased interface impedance caused by massive Li dendrites and "dead" Li.

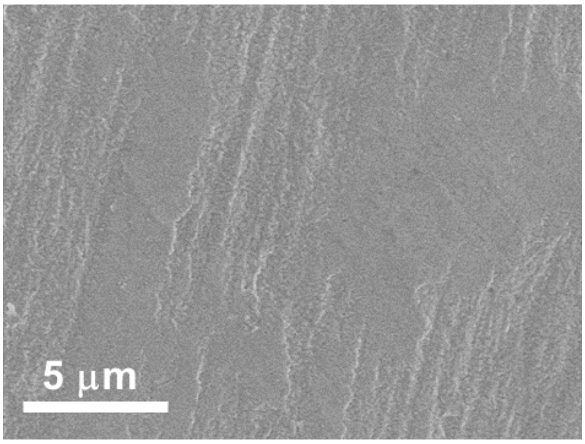

Figure S5. SEM image of the Cu foil before use.

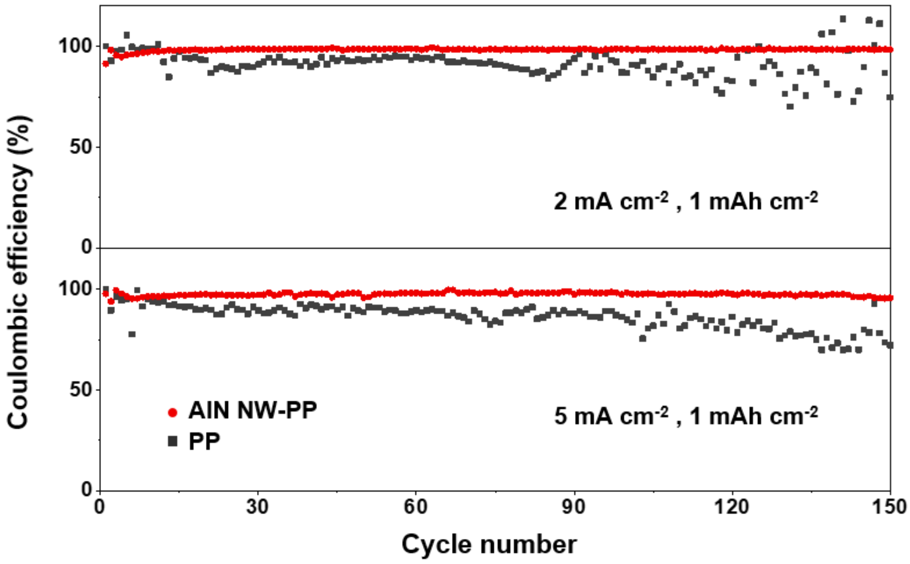

Figure S6. Coulombic efficiencies of Li|Cu cells with AlN NW-PP or PP separator at the marked current densities. The capacity is 1 mAh cm^{-2}.

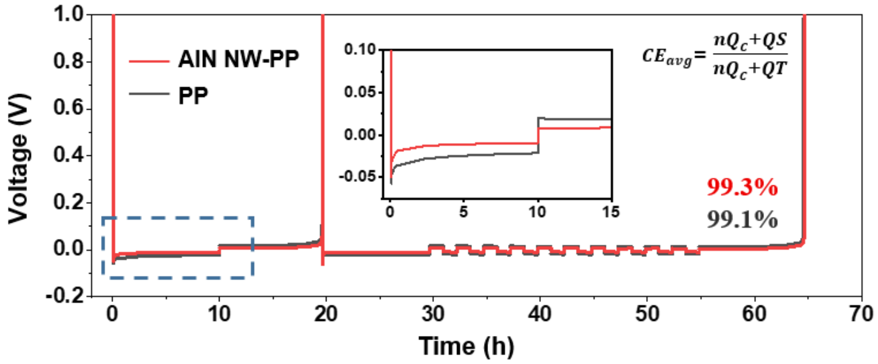

Figure S7. Potential profiles of Li|Cu cells with AlN NW-PP or PP separator to measure the average CE.

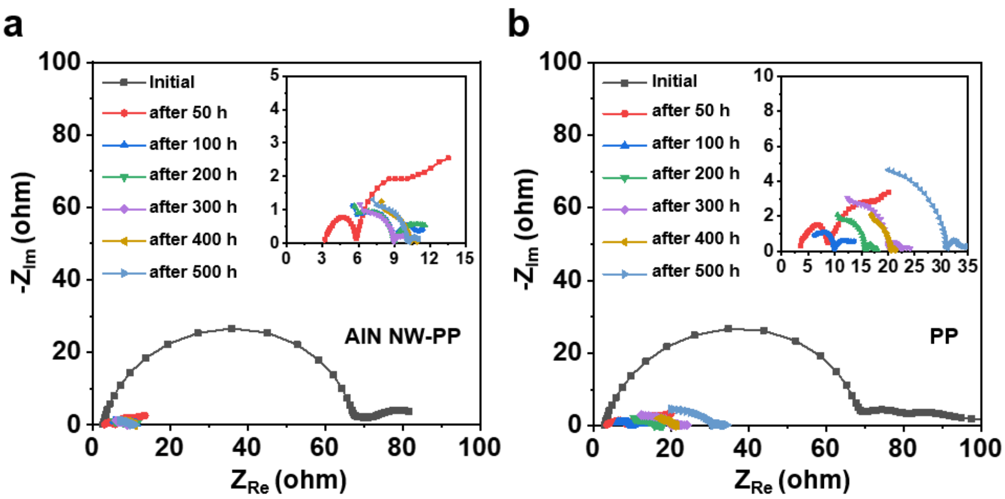

Figure S8. EIS spectra of symmetric Li|Li cells with different separators. (a) AlN NW-PP, (b) PP.

At the initial stage, the two symmetric Li|Li cells have close charge transfer resistances (R_{ct}). Once cycled, R_{ct} of the Li|AlN NW-PP|Li is lower than that of the Li|PP|Li after the same cycles, and the increasing rate of the resistance with the cycle number is much slower for the former than the latter, which indicates the inhibited Li dendrites growth and less electrolyte depletion for the former. The sharp decrease after the first cycle and the subsequent increase of R_{ct} correspond to the activation of batteries and accumulation of "dead" Li, respectively.

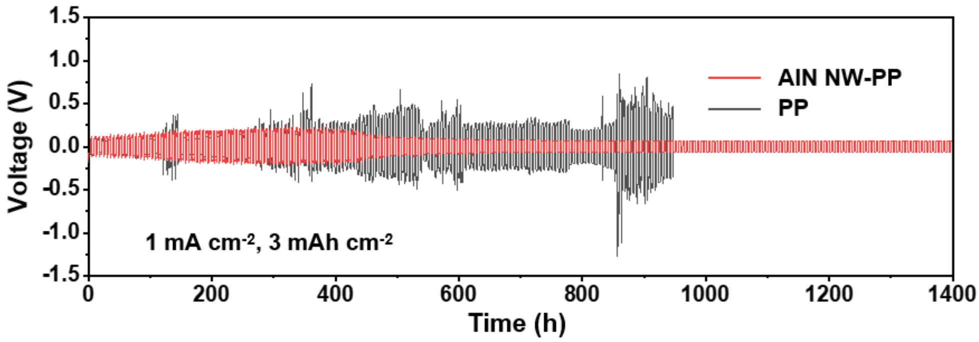

Figure S9. Galvanostatic cycling of symmetric Li|Li cells in commercial carbonate electrolyte (1 mol L^{-1} $LiPF_6$ in EC/DMC (1/1, v/v)).

Li|AlN NW-PP|Li: Small plateau voltage and long-term cycling stability.

Li|PP|Li: Large voltage fluctuation and quick failure.

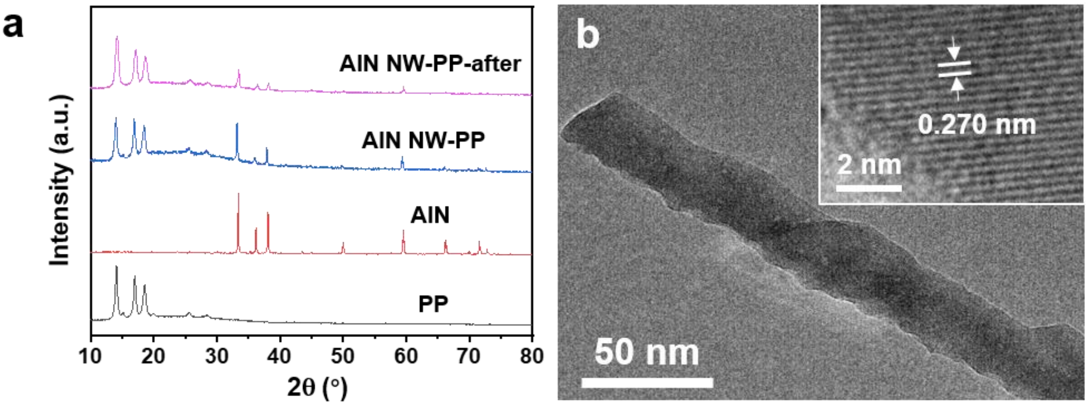

Figure S10. Characterizations of PP, AlN nanowires, AlN NW-PP, and AlN NW-PP after 600 h cycling. (a) XRD patterns. (b) TEM image of AlN nanowires after cycling. The inset in (b) is the HRTEM image.

The AlN NW-PP separators before and after cycling have identical XRD patterns (Figure S8a), and the TEM image shows the good crystallization of the AlN nanowires after cycling (Figure S8b). These results indicate the excellent electrochemical stability of AlN NW.

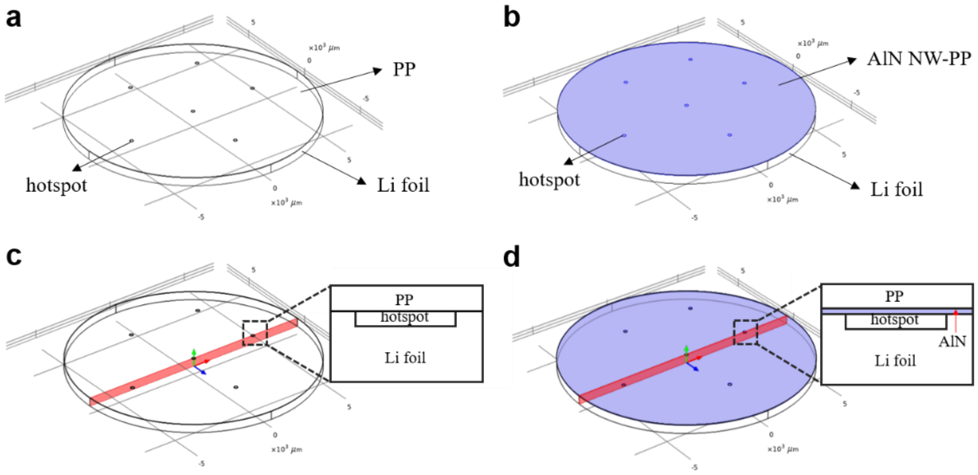

Figure S11. The models constructed in COMSOL Multiphysics software. (a,b) The 3D modeling diagrams for the cells with PP (a) or AlN NW-PP (b). (c,d) The 2D cross-sections for observing temperature and Li^+ distributions in the cells with PP (c) or AlN NW-PP (d).

A three-dimensional (3D) spatial model with Li-metal anode (500 μm thick, 7 mm in radius), pristine PP separator (25 μm thick, 7 mm in radius), and AlN layer (6.5 μm thick, 7 mm in radius) was constructed. Li-ions are concentrated at the top of the separator and transferred to the surface of the Li-metal anode by the packed electrolytes. Six heat sources (15 μm in thickness, 100 μm in radius) are created on the Li foil to represent the hotspots generated during cycling.

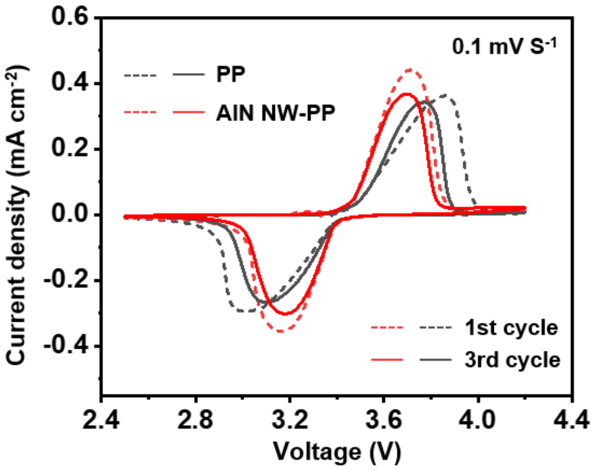

Figure S12. The cyclic voltammetry curves of the Li|AlN NW-PP|$LiFePO_4$ and Li|PP|$LiFePO_4$ cells.

The Li|AlN NW-PP|$LiFePO_4$ cell exhibits a smaller potential gap between the anodic and cathodic peaks than that of the Li|PP|$LiFePO_4$ cell, suggesting the mitigated polarization for the former. The CV curve of the Li|AlN NW-PP|$LiFePO_4$ is similar to that of the Li|PP|$LiFePO_4$ without new redox peaks. This result indicates that no side reaction is introduced with the AlN modification.

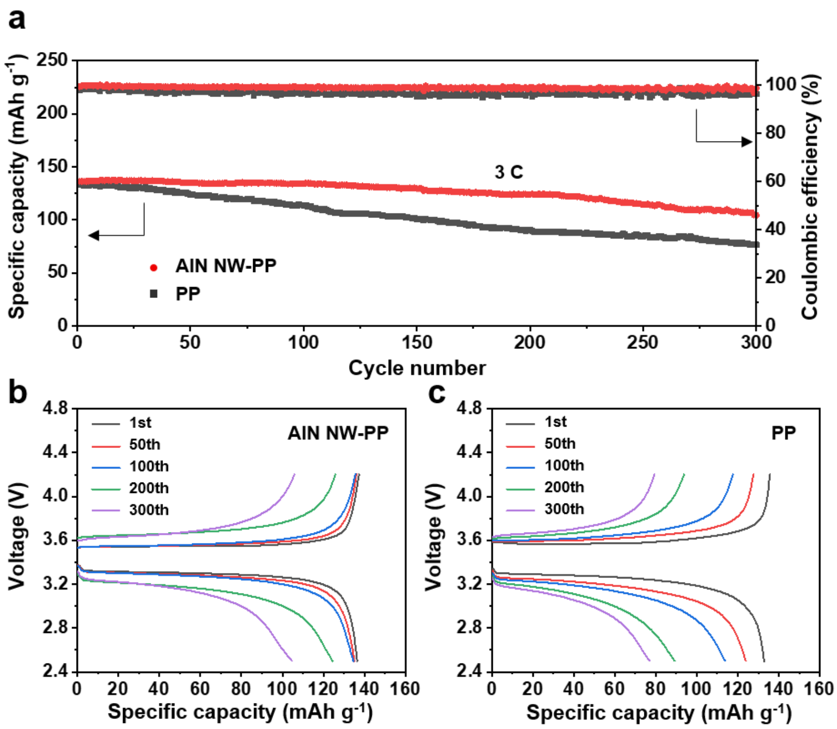

Figure S13. Electrochemical performances of Li|AlN NW-PP|$LiFePO_4$ and Li|PP|$LiFePO_4$ cells under high current density (3 C). (a) Long-term cycling stability. (b,c) Charge/discharge curves of Li|AlN NW-PP|$LiFePO_4$ (b) and Li|PP|$LiFePO_4$ (c).

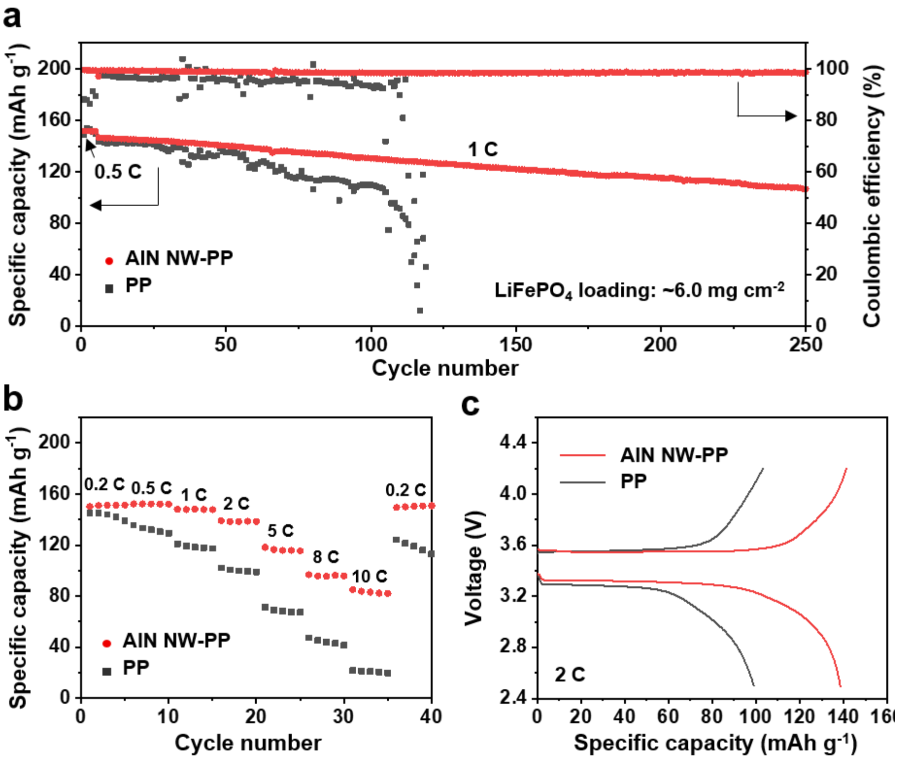

Figure S14. Electrochemical performances of Li|AlN NW-PP|$LiFePO_4$ and Li|PP|$LiFePO_4$ cells with the $LiFePO_4$ loading of 6 mg cm^{-2}. (a) Cycling stability. (b) Rate performance. (c) Charge/discharge curves at 2 C.

The Li|AlN NW-PP|$LiFePO_4$ cell still exhibited more stable long-term cycling and higher rate performance than the Li|PP|$LiFePO_4$ cell.

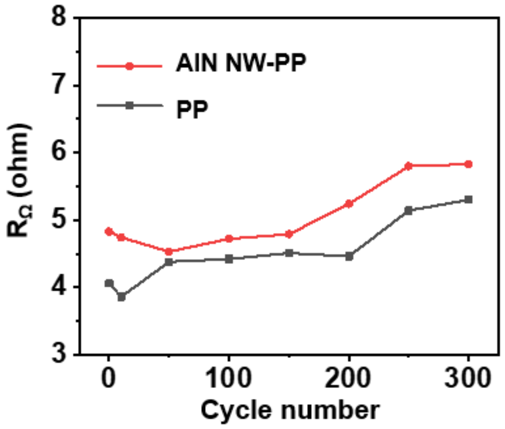

Figure S15. Variation of R_Ω versus cycle number for Li|$LiFePO_4$ cells.

The R_Ω values of the Li|AlN NW-PP|$LiFePO_4$ cell are slightly higher than that of the Li|PP|$LiFePO_4$ cell, which may result from the increased ion transport length between anode and cathode due to the insertion of AlN NW shield on PP.

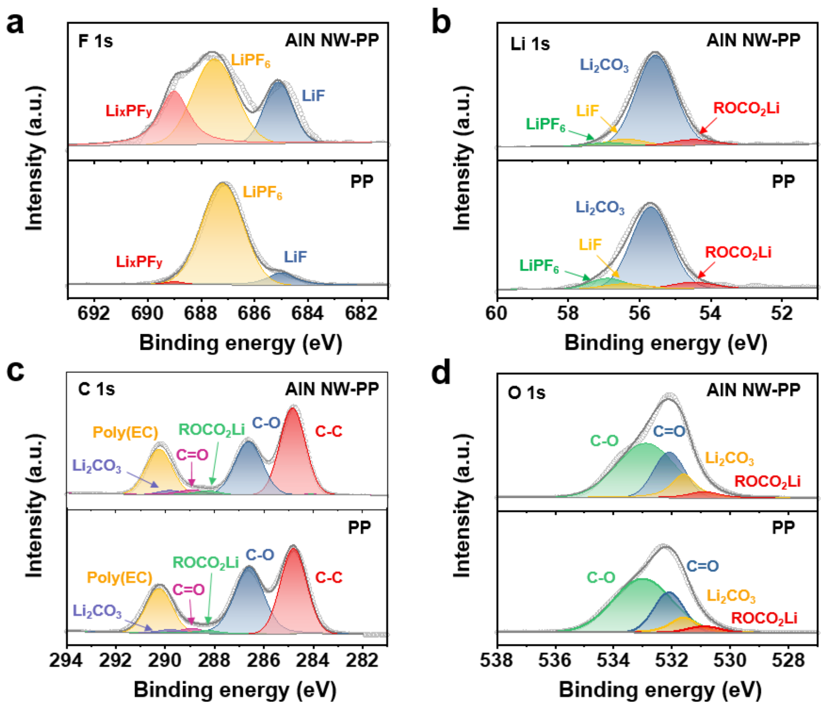

Figure S16. XPS spectra of Li anodes from Li|$LiFePO_4$ cells after 20 cycles. (a) F 1s; (b) Li 1s; (c) C 1s; (d) O 1s.

The XPS spectra indicate the SEI film in situ formed via the reactions between electrolyte and Li anodes is mainly composed of the inorganic (Li_2CO_3, LiF, and Li_xPF_y) and organic ($ROCO_2Li$) species. More LiF and Li_xPF_y species were detected on the cycled Li anode of the Li|AlN NW-PP|$LiFePO_4$ than the Li|PP|$LiFePO_4$.

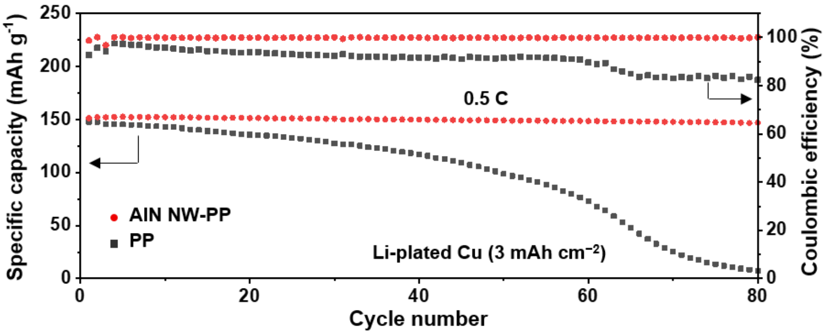

Figure S17. Cycling performances of $LiFePO_4$|Li-plated Cu cells with AlN NW-PP or PP separator. The Cu foil was plated with 3 mAh cm^{-2} of Li in advance.

The $LiFePO_4$|Li-plated Cu cell with AlN NW-PP exhibits a much better cycling performance than the cell with PP.

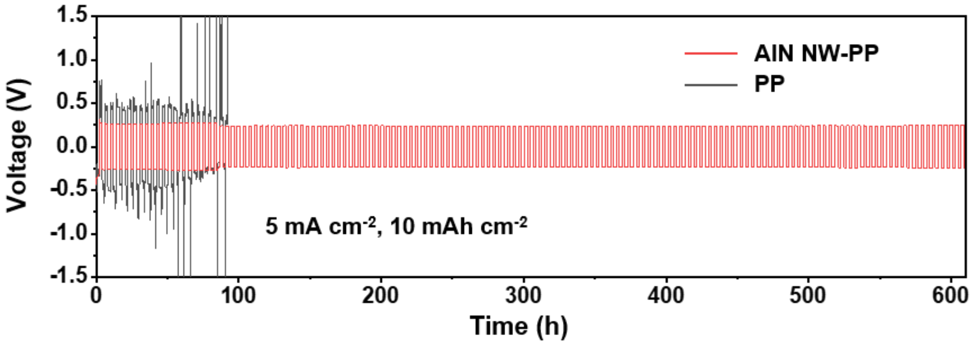

Figure S18. Electrochemical performances of symmetric Na|Na cells with AlN NW-PP or PP separator at 5 mA cm^{-2}, 10 mAh cm^{-2}.

The Na|AlN NW-PP|Na cell keeps stable overpotential over 600 h at a high current density of 5 mA cm^{-2} with a capacity of 10 mAh cm^{-2}. In contrast, the Na|PP|Na cell shows larger polarization and shorter lifespan than the former, which should be attributed to the uneven deposition and severe dendrite growth. This difference is similar to the case for symmetric Li|Li cells with or without AlN NW shield.

Table S1. Comparison of the electrochemical performances for the Li|Cu cells and the symmetric Li|Li cells in ether-based electrolytes.

Samples	Li|Cu cells				Symmetric Li|Li cells		
	Current ($mA\ cm^{-2}$)	Capacity ($mAh\ cm^{-2}$)	Cycles	CEs	Current ($mA\ cm^{-2}$)	Capacity ($mAh\ cm^{-2}$)	Time (hour)
**Designing the composite separators**							
AlN NW (this study)	1.0<br>2.0<br>5.0	1.0<br>1.0<br>1.0	250<br>150<br>150	98.5%<br>98.4%<br>95.7%	3.0<br>20.0<br>50.0<br>80.0	3.0<br>3.0<br>25.0<br>80.0	5000<br>8000<br>5000<br>1000
PB/rGO[5]	1.0	1.0	200	98.0%	1.0	1.0	600
g-C_3N_4[6]	1.0	1.0	900	99.0%	1.0	1.0	1000
PLLZ[7]	1.0	1.0	300	97.5%	1.0<br>1.0	1.0<br>3.0	1000<br>600
GO-g-PAM[8]	1.0	1.0	150	98.0%	2.0<br>10.0<br>20.0	1.0<br>1.0<br>5.0	2600<br>2100<br>1900
MIL-125-Ti MOF[9]	2.0	2.0	170	98.1%	2.0<br>4.0	2.0<br>4.0	500<br>500
AlF_3/PVDF-HFP[10]	1.0	1.0	120	98.0%	3.0<br>3.0<br>5.0	1.0<br>3.0<br>1.0	600<br>400<br>200
LiAl LDH[11]	1.0	1.0	200	98.0%	1.0<br>20.0	1.0<br>5.0	1600<br>3000
GFNs-PVDF[12]	1.0	1.0	120	~98.0%	2.0<br>5.0<br>20.0	1.0<br>1.0<br>3.0	1200<br>1100<br>300
Zn[13]	1.0	1.0	200	~97.0%	1.0<br>3.0	1.0<br>1.0	1000<br>650
DLC[14]	0.5	0.5	450	98.0%	3.0<br>5.0	3.0<br>5.0	4500<br>4000
**Constructing the stable Li hosts**							
rGO[15]	1.0<br>2.0	1.0<br>2.0	300<br>300	99.0%<br>98.0%	1.0	1.0	500
mPPy-GO[16]	0.5	0.5	1000	98.2%	1.0<br>5.0	1.0<br>1.0	1100<br>400
quicksand-like LMA[17]	/	/	/	/	1.0<br>20.0	1.0<br>8.0	1000<br>400
CNT sponge macrofilm[18]	1.0<br>10.0	0.5<br>0.5	100<br>300	~97.0%<br>~86.0%	2.0<br>20.0<br>40.0	1.0<br>10.0<br>2.0	300<br>300<br>2000
**Optimizing the electrolyte**							
Fibroin[19]	1.0	1.0	100	98.0%	3.0	1.0	1000
Graphene quantum dots[20]	1.0	1.0	150	97.4%	40.0<br>60.0	20.0<br>60.0	1200<br>1100

Note:
CEs: Coulombic efficiencies; **PB:** Prussian blue; **rGO**: reduced graphene oxide;
PLLZ: composite of polyvinylidene fluoride (PVDF) and $Li_{6.4}La_3Zr_{1.4}Ta_{0.6}O_{12}$(LLZTO);
GO-g-PAM: polyacrylamide-grafted graphene oxide nanosheets;
PVDF-HFP: Poly(vinylidene-*co*-hexafluoropropylene); **LiAl LDH**: LiAl layered double hydroxide;
GFNs: graphite fluoride nanosheets; **DLC**: diamond-like carbon;
mPPy-GO: mesoporous polypyrrole on graphene oxide; **LMA**: lithium metal anode; **CNT**: carbon

nanotube

References

[1] Q. Wu, Z. Hu, X. Wang, Y. Chen, *J. Phys. Chem. B* **2003**, *107*, 9726.

[2] Z. Hao, Y. Wu, Q. Zhao, J. Tang, Q. Zhang, X. Ke, J. Liu, Y. Jin, H. Wang, *Adv. Funct. Mater.* **2021**, *31*, 2102938.

[3] B. D. Adams, J. Zheng, X. Ren, W. Xu, J.-G. Zhang, Adv. Energy Mater. **2018**, *8*, 1702097.

[4] C. Zhang, L. Dong, N. Zheng, H. Zhu, C. Wu, F. Zhao, W. Liu, *Energy Storage Mater.* **2021**, *37*, 296.

[5] X. Wu, N. Liu, Z. Guo, M. Wang, Y. Qiu, D. Tian, B. Guan, L. Fan, N. Zhang, *Energy Storage Mater.* **2020**, *28*, 153.

[6] Y. Guo, P. Niu, Y. Liu, Y. Ouyang, D. Li, T. Zhai, H. Li, Y. Cui, *Adv. Mater.* **2019**, *31*, 1900342.

[7] H. Huo, X. Li, Y. Chen, J. Liang, S. Deng, X. Gao, K. Doyle-Davis, R. Li, X. Guo, Y. Shen, C.-W. Nan, X. Sun, *Energy Storage Mater.* **2020**, *29*, 361.

[8] C. Li, S. Liu, C. Shi, G. Liang, Z. Lu, R. Fu, D. Wu, *Nat. Commun.* **2019**, *10*, 1363.

[9] Y. Zhong, F. Lin, M. Wang, Y. Zhang, Q. Ma, J. Lin, Z. Feng, H. Wang, *Adv. Funct. Mater.* **2020**, *30*, 1907579.

[10] L. Wang, S. Fu, T. Zhao, J. Qian, N. Chen, L. Li, F. Wu, R. Chen, *J. Mater. Chem. A* **2020**, *8*, 1247.

[11] Q.-K. Lei, Q. Zhang, X.-Y. Wu, X. Wei, J. Zhang, K.-X. Wang, J.-S. Chen, *Chem Eng J* **2020**, *395*, 125187.

[12] J. Xiao, P. Zhai, Y. Wei, X. Zhang, W. Yang, S. Cui, C. Jin, W. Liu, X. Wang, H. Jiang, Z. Luo, X. Zhang, Y. Gong, *Nano Lett.* **2020**, *20*, 3911.

[13] L. Lin, F. Liu, X. Yan, Q. Chen, Y. Zhuang, H. Zheng, J. Lin, L. Wang, L. Han, Q. Wei, Q. Xie, D.-L. Peng, *Adv. Funct. Mater.* **2021**, 2104081.

[14] Z. Li, M. Peng, X. Zhou, K. Shin, S. Tunmee, X. Zhang, C. Xie, H. Saitoh, Y. Zheng, Z.

Zhou, Y. Tang, *Adv. Mater.* **2021**, *33*, 2100793.

[15] N. Li, K. Zhang, K. Xie, W. Wei, Y. Gao, M. Bai, Y. Gao, Q. Hou, C. Shen, Z. Xia, B. Wei, *Adv. Mater.* **2020**, *32*, 1907079.

[16] H. Shi, J. Qin, K. Huang, P. Lu, C. Zhang, Y. Dong, M. Ye, Z. Liu, Z.-S. Wu, *Angew. Chem. Int. Ed.* **2020**, *59*, 12147.

[17] Y. Zhang, Z. Han, Z. Huang, C. Zhang, C. Luo, G. Zhou, W. Lv, Q.-H. Yang, *ACS Energy Lett.* **2021**, *6*, 3761.

[18] Z. Y. Wang, Z. X. Lu, W. Guo, Q. Luo, Y. H. Yin, X. B. Liu, Y. S. Li, B. Y. Xia, Z. P. Wu, *Adv. Mater.* **2021**, *33*, 2006702.

[19] T. Wang, Y. Li, J. Zhang, K. Yan, P. Jaumaux, J. Yang, C. Wang, D. Shanmukaraj, B. Sun, M. Armand, Y. Cui, G. Wang, *Nat. Commun.* **2020**, *11*, 5429.

[20] L. Ye, M. Liao, X. Cheng, X. Zhou, Y. Zhao, Y. Yang, C. Tang, H. Sun, Y. Gao, B. Wang, H. Peng, *Angew. Chem. Int. Ed.* **2021**, *60*, 17419.
